# A Rare Case of Acute Cord Compression From Spinal Myeloid Sarcoma: A Complication of Acute Myeloid Leukemia

**DOI:** 10.7759/cureus.9502

**Published:** 2020-08-01

**Authors:** Mukesh Kumar, FNU Sonia, Syeda M Hamadani, Syed A Abbas

**Affiliations:** 1 Internal Medicine, Montefiore Medical Center, Wakefield Campus, Bronx, USA; 2 Internal Medicine, Albert Einstein College of Medicine, Bronx, USA; 3 Internal Medicine, Fatima Memorial Hospital College of Medicine and Dentistry, Lahore, PAK

**Keywords:** acute myeloid leukemia (aml), myeloid sarcoma

## Abstract

Acute myeloid leukemia (AML) is the most common malignancy in the acute leukemia category. AML is a very aggressive cancer with high mortality. The most common presentations include pancytopenia, bleeding, and recurrent infections. Unlike lymphoma, it rarely presents as a mass. Myeloid sarcoma is a peripheral collection of myeloid cells. Myeloid sarcoma most commonly involves the skin and gingival tissue and rarely it affects the central nervous system. Myeloid sarcoma involving the central nervous system is associated with high mortality. We present a patient with AML which evolved from myelofibrosis presented with acute spinal cord compression and found to have myeloid sarcoma involving the thoracic spinal cord. Despite acute radiation therapy, the patient could not recover her neurological function and passed away shortly after the diagnosis. We discuss the importance of early recognition of the complication due to myeloid sarcoma and treatment with neurosurgical intervention just like other mass causing acute cord compression.

## Introduction

Primary myelofibrosis transformation into acute myeloid leukemia (AML) carries a very poor prognosis [1-2]. AML most commonly presents with symptoms related to complications of pancytopenia including weakness and easy fatigability, infections, and hemorrhagic findings such as gingival bleeding, ecchymosis, or epistaxis. A small percentage of patients can present with prominent extramedullary diseases like myeloid sarcoma, myeloblastoma, or chloroma. Most commonly, extramedullary involvement manifests as either cutaneous or gingival [3-4]. We present a patient who developed acute cord compression from myeloid sarcoma at the thoracic spine.

## Case presentation

A 64-year-old woman with myelofibrosis (JAK2 V617F positive) on ruxolitinib for many years presented with abdominal pain and generalized fatigue. Initial evaluation revealed hepatosplenomegaly with splenic and hepatic infarcts. Bone marrow biopsy revealed 20% blasts with positive CD34 and CD117, suggestive of transformation to AML. The patient was offered chemotherapy but the patient refused. Later after two months she was admitted with worsening abdominal pain and was found to have worsening hepatosplenomegaly; peripheral flow cytometry revealed 38% blasts. On Day 5 of her admission, she complained of loss of power in lower extremities with bowel and urinary incontinence. MRI thoracic and lumbar revealed posterior epidural soft tissue/tumor in the thoracic spine at T3-T4, T5/T6/T7, and T9-T10 with resultant cord compression. Due to the critical condition of the patient and based on the oncologist’s suggestion that is most likely myeloid sarcoma, biopsy was deferred and the patient underwent emergent radiotherapy for thoracic lesion and started on chemotherapy. Unfortunately, she did not recover her neurological function. She eventually was transferred to hospice and expired soon after.

**Figure 1:**
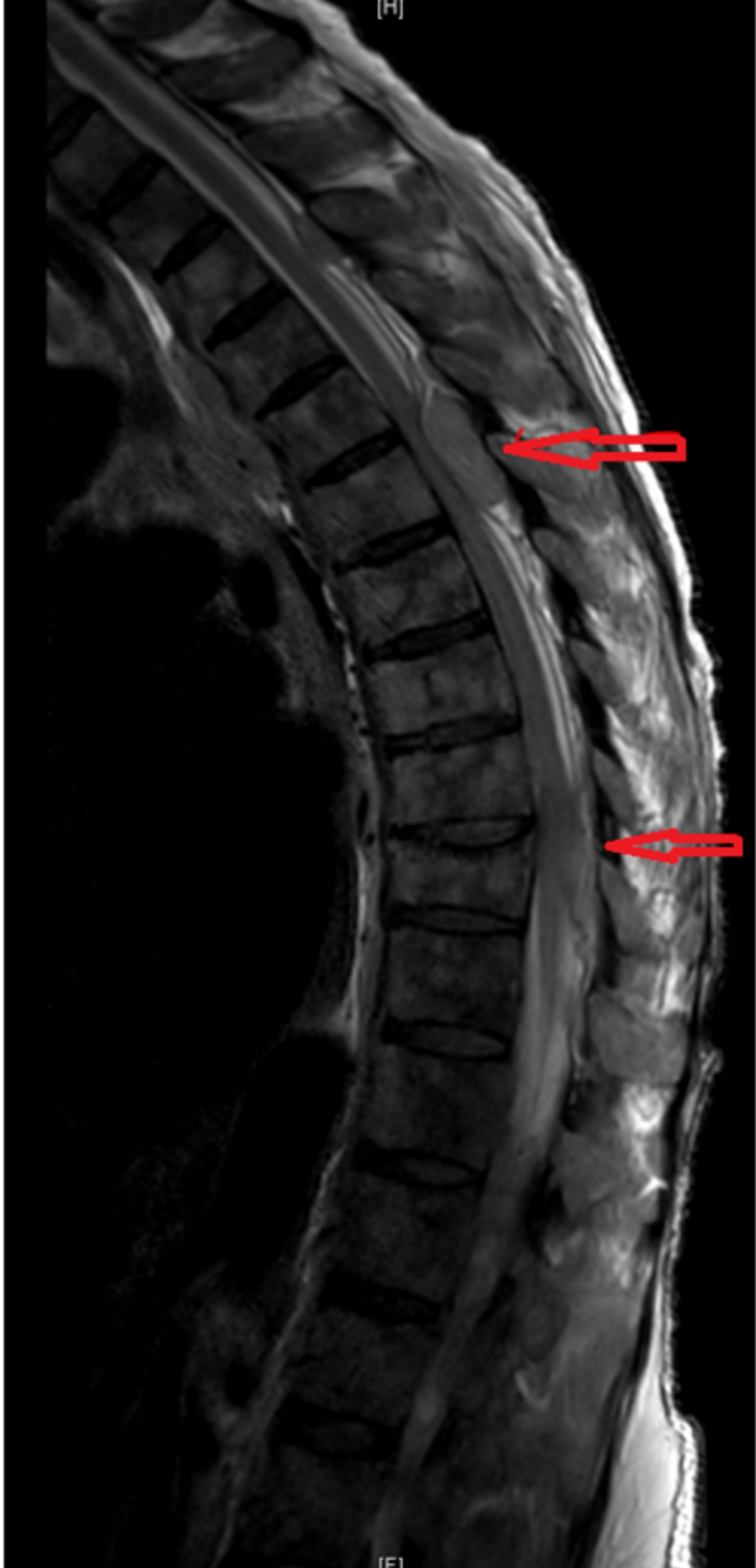
MRI of thoracic spine-posterior epidural soft tissue in the thoracic spine at T3-T4, T5/T6/T7, and T9-T10 with resultant cord compression most prominent at T6 and T9.

## Discussion

Myeloid sarcoma is an extramedullary proliferation of blasts of leukemic cells that leads to disruption of the normal architecture of tissue in which it deposits. Myeloid sarcoma in patients with AML is associated with poor prognosis [5]. We describe a case where a patient had AML transformed from myelofibrosis who developed acute neurological symptoms of cord compression and was found to have soft tissue/tumor on the epidural surface of T6-T9. Though the patient did not have a biopsy of the mass the history of her disease, the acuteness of symptom development, and lack of prior vertebral pathology were strongly suggestive of myeloid sarcoma of epidural space. Diagnosis of mass effect is rarely thought in patients with AML as AML rarely presents as a mass. However, rarely it can present as a mass in some cases and most common sites of involvement are extramedullary such as skin or gingival surface. Myeloid sarcoma involving tissue such as skin, or gingival surface has lower mortality as compared to tissue such as the brain and spinal cord. There is a case series of 21 patients demonstrating spine sarcoma from AML mortality was 53% in a median follow up of nine months [6].

Early neurosurgical intervention in disease causing acute cord compression from a mass is associated with high survival compared to without intervention. Our patient received early radiotherapy as it was thought epidural myeloid sarcoma has high mitotic activity but early neurosurgery intervention should have been considered in such a patient as it can change outcomes. Our patient did not recover her neurological function and was sent to hospice and she died soon after. It is also important to note that patients who present with an acute neurological compromise with known liquid malignancy and a focused neurological evaluation should be considered with neurosurgery on board. 

## Conclusions

Liquid malignancy such as AML, acute lymphoblastic leukemia, chronic myeloid leukemia, chronic lymphoblastic leukemia rarely present as a mass effect. A patient with known malignancy even if it is liquid malignancy as in our case of AML when present with acute life-threatening neurological symptoms such as new-onset weakness, altered mental status, sudden loss lower extremities power should be worked up emergently and if possible a neurosurgery evaluation should be considered for to avoid life-threatening complications and improve patient outcomes.
